# Supplementary material for: Compression of Structured High-Throughput Sequencing Data
Source: PLoS One. 2013 Nov 18;8(11):e79871. doi: 10.1371/journal.pone.0079871 (PMC3832420; doi:10.1371/journal.pone.0079871)
Supplement: File S1 — Supporting Tables and Figures. This file contains Table S1, Table S2 and Figure S1. Table S1: Details of the Benchmark Datasets; Table S2: General Compression Benchmark relative to GZip; Figure S1: Structured data schemas and Large Collection Storage Protocol. (PDF) [file pone.0079871.s001.pdf]

Supporting information for:

Compression of structured high-throughput sequencing data

Fabien Campagne[1,2]*, Kevin C. Dorff[1], Nyasha Chambwe[1,2], James T. Robinson[3], Jill P. Mesirov[3].

[1]The HRH Prince Alwaleed Bin Talal Bin Abdulaziz Alsaud Institute for Computational Biomedicine. [2]Department of Physiology and Biophysics, The Weill Cornell Medical College, New York, NY, USA. [3]Broad Institute of Massachusetts Institute of Technology and Harvard, Cambridge, MA, USA.

*Correspondence to: Fabien Campagne, fac2003@campagnelab.org.

Supplementary Materials

- Tables S1-S2
- Figures S1

Table S1. Details of the Benchmark Datasets

Kind	BAM Tag	Paired-end	Spliced	Accession Code	Range	BAM file size	#reads	% mapped	Organism	URL
Exome	HZFWPTI	Yes	No	NA12340	chr11	550 MB	6,787,665	98.97%	human	[link]
Exome	UANMNXR	Yes	No	NA20766	ch11	501 MB	6,139,330	99.19%	human	[link]
RNA-Seq	MYHZZJH	No	Yes	NA18853	all	2.8 GB	19,365,426	98.64%	human	
RNA-Seq	ZHUUJKS	No	Yes	NA19172	all	1.5 GB	18,749,217	97.66%	human	
RNA-Seq	EJOYQAZ	Yes	Yes		whole genome	904 MB	15,693,880	100.00%	human	[link]
RRBS	JRODTYG	No	No	GSM675439	representative genome	1.1 GB	41,186,902	81.68%	mouse	[link]
Methyl-Seq	ZVLRRJH	No	No	GSM721194	whole genome	1.6 GB	19,999,974	100.00%	human	[link]
WGS	XAAOBVT	Yes	No	ERP000765	FC1, ~ first 20M reads	1.4 GB	19,999,953	100.00%	human	[link]
WGS	UCCWRUX	Yes	No	ERP000765	FC2, ~ first 20M reads	1.4 GB	19,999,953	98.17%	human	[link]
WGS	HENGLIT	Yes	No	SRR065390	First 30 million reads	1.7 GB	30,000,000	93.06%	c Elegans	[link]

Table S2. General Compression Benchmark relative to GZip

Storage efficiency is calculated as the ratio of the size of compressed data with each method (H, H+T or H+T+D) vs GZip compressed data size, expressed as a percentage. A storage efficiency of 50% indicates that the specific method compressed the dataset to half the size of method GZip compression. Compression/Decompression time ratios measure the ratio of the time it takes a specific method to compress/decompress a dataset compared to the time it takes the GZip compression method for the same dataset. A ratio of 200% indicates that the specific method is twice slower than GZip. See Fig. 1 for a description of the H, H+T and H+T+D methods.

	Storage Efficiency			Compression Times			Decompression Times		
**Sample ID**	**H**	**H+T**	**H+T+D**	**H**	**H+T**	**H+T+D**	**H**	**H+T**	**H+T+D**
HZFWPTI	42%	39%	29%	209%	226%	200%	209%	185%	209%
UANMNXR	42%	39%	29%	197%	203%	194%	200%	169%	194%
MYHZZJH	25%	21%	19%	328%	241%	282%	281%	201%	294%
ZHUUJKS	27%	23%	21%	290%	266%	252%	271%	190%	250%
EJOYQAZ	48%	41%	38%	201%	205%	206%	252%	209%	266%
JRODTYG	109%	59%	50%	312%	252%	286%	389%	165%	186%
ZVLRRJH	83%	46%	33%	268%	223%	265%	533%	270%	289%
XAAOBVT	62%	38%	31%	181%	189%	208%	213%	183%	245%
UCCWRUX	54%	35%	27%	234%	235%	205%	241%	197%	229%
HENGLIT	43%	42%	32%	256%	287%	285%	320%	282%	339%
**Average:**	**54%**	**38%**	**31%**	**248%**	**233%**	**238%**	**291%**	**205%**	**250%**

Supplementary Figures

Fig. S1. Structured data schemas and Large Collection Storage Protocol.

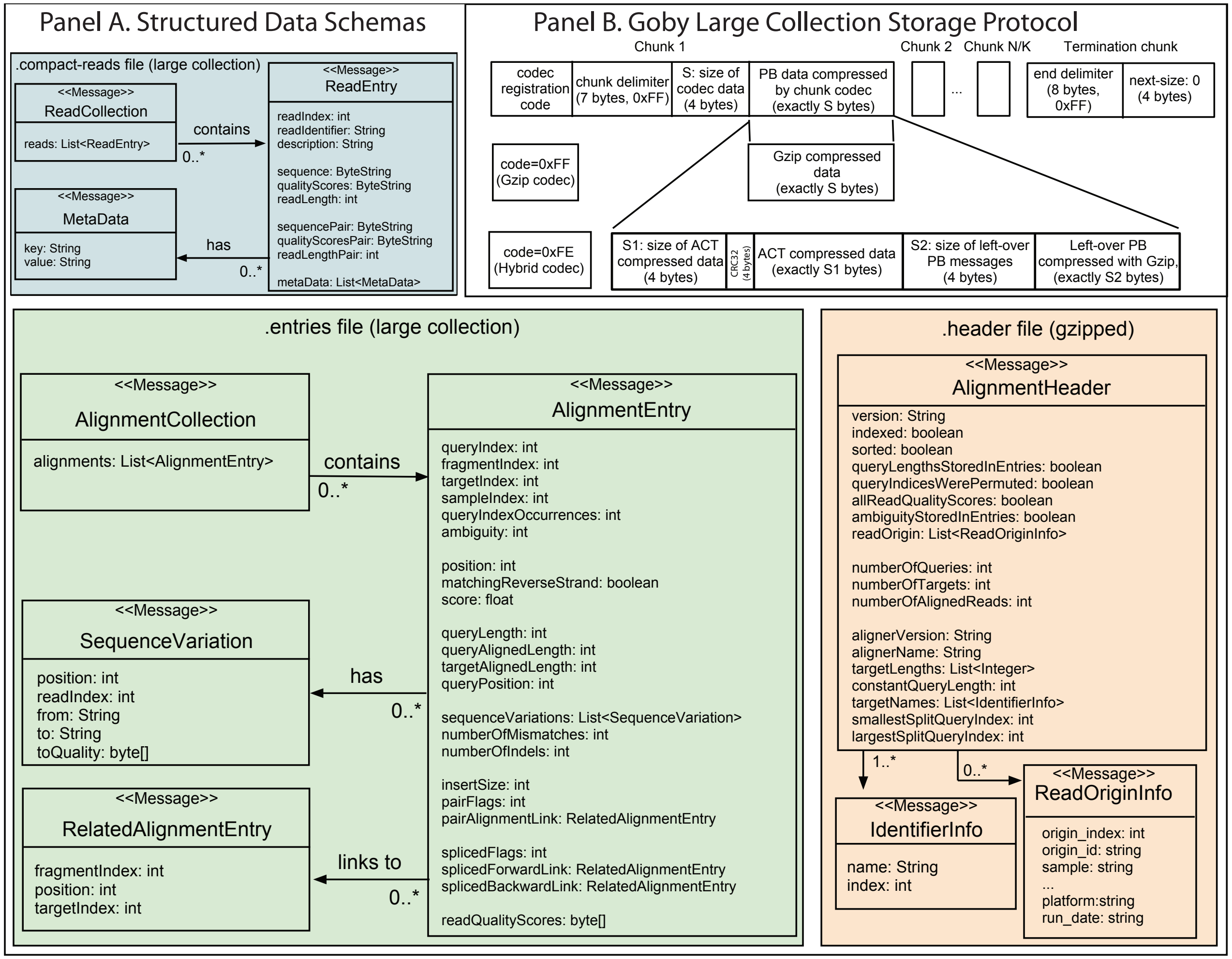

Figure S1. Structured data schemas and Large Collection Storage Protocol. (**A**) Describes the data schemas that we have devised to represent HTS reads and alignments. These schemas are described following the Unified Modeling Language conventions. Briefly, data are organized as Protocol Buffer messages (equivalent of data structures), which can contain data fields of primitive types or other messages. We store reads as collections of ReadEntry messages and alignments as collections of AlignmentEntry messages. References between messages are represented with integer indices. For instance, the RelatedAlignmentEntry message is used to link two alignment entries to represent paired or spliced alignments. (**B**) Describes how large collections of messages are stored piecewise in chunks of compressed PB data. The Goby Large Collection Storage Protocol (GLCSP) provides a plugin mechanism to define new PB compression/decompression approaches (codecs). The Hybrid codec compresses one part of a collection with the ACT approach (H, H+T or H+T+D variants) and the reduced messages remaining after ACT compression with GZip.
